# MiR-371-5p Facilitates Pancreatic Cancer Cell Proliferation and Decreases Patient Survival

**DOI:** 10.1371/journal.pone.0112930

**Published:** 2014-11-20

**Authors:** De He, Huilai Miao, Yumin Xu, Longhui Xiong, Yi Wang, Hongxia Xiang, Heng Zhang, Zhiyong Zhang

**Affiliations:** 1 Department of General Surgery, Affiliated Baoan Hospital of Southern Medical University, Shenzhen, China; 2 Department of Hepatobiliary Surgery, Affiliated Hospital of Guangdong Medical College, Zhanjiang, Guangdong, China; 3 Department of Medicine, College of Clinical Science of China, Three Gorges University, Yichang, Hubei, China; 4 Department of Surgery, Robert Wood Johnson Medical School University Hospital, Rutgers Unversity, New Brunswick, New Jersey, United States of America; The University of Hong Kong, China

## Abstract

**Background:**

microRNAs (miRNAs) play a critical role in tumorigenesis, either as a tumor suppressor or as an oncogenic miRNA, depending on different tumor types. To date, scientists have obtained a substantial amount of knowledge with regard to miRNAs in pancreatic cancer. However, the expression and function of miR-371-5p in pancreatic cancer has not been clearly elucidated. The aim of this study was to investigate the roles of miR-371-5p in pancreatic cancer and its association with the survival of patients with pancreatic cancer.

**Methods:**

The expression of miR-371-5p was examined in pancreatic duct adenocarcinoma (PDAC) and their adjacent normal pancreatic tissues (ANPT) or in pancreatic cancer cell lines by qRT-PCR. The association of miR-371-5p expression with overall survival was determined. The proliferation and apoptosis of SW-1990 and Panc-1 cells, transfected with miR-371-5p mimics or inhibitor, were assessed using MTT assay and flow cytometry, respectively. The tumorigenicity was evaluated via mice xenograft experiments. miR-371-5p promoter interactions were analyzed by chromatin immunoprecipitation assays (ChIP). Protein expression was analyzed by Western blot.

**Results:**

The expression level of miR-371-5p was dramatically upregulated in clinical PDAC tissues compared with ANPT. Patients with high miR-371-5p expression had a significantly shorter survival than those with low miR-371-5p expression. The in vitro and in vivo assays showed that overexpression of miR-371-5p resulted in cell proliferation and increased tumor growth, which was associated with inhibitor of growth 1 (ING1) downregulation. Interestingly, we also found that ING1, in turn, inhibited expression of miR-371-5p in the promoter region.

**Conclusions:**

our study demonstrates a novel ING1-miR-371-5p regulatory feedback loop, which may have a critical role in PDAC. Thus miR-371-5p can prove to be a novel prognostic factor and therapeutic target for pancreatic cancer treatment.

## Introduction

PDAC is a highly malignant phenotype characterized by rapid progression, early metastasis, and a limited response to radiotherapy and chemotherapy. Despite more than 10 years of FDA-approved therapeutic regimens and great improvements in medical care, no significant effect on PDAC patient survival has been observed [1]. Now it is the fourth leading cause of cancer-related deaths in the U.S. with a 5% 5-year survival rate annually [2]. Therefore, there is an urgent need to identify biomarkers that will promote early diagnosis and allow personalized treatment strategies for patients at high risk of PDAC.

The development and progression of PDAC are a complex process, involving the deregulation of multiple genes that are essential to cell biological processes. Transcription factors (TFs) and miRNAs are prominent regulators for gene expression [3]. In the past 10 years, miRNAs are particularly important in nearly all tumor development studies because they can be targets of genomic lesions, controlled by classic tumor signals, and they themselves present as a class of oncogenes or tumor suppressors [4]. Recently, miRNAs have been suggested to be closely associated with PDAC tumorigenesis [5].

miRNAs are small (21–24 nt) non-coding RNA molecules that regulate gene expression by binding loosely complementary sequences in the 3′-untranslated region of target mRNAs to repress translation or induce mRNA cleavage [6]. The discovery of miRNAs has shed new insights regarding the regulation of cell proliferation, differentiation, apoptosis and senescence, and has also contributed to patient management, including diagnosis and treatment [7].

Although previous studies have clarified the roles of many miRNAs in pancreatic cancer [8], no studies have addressed the role of miR-371-5p. Recent studies demonstrated that miR-371-5p was significantly elevated in gastric cancer (GC) patients and regulated hepatocellular carcinoma (HCC) cell proliferation by accelerating the G1/S transition [9], [10]. Especially, further validation indicated that serum miR-371-5p, as a novel biomarker, had strong potential in discriminating GC patients from healthy controls [9]. We therefore hypothesize that miR-371-5p may also be a novel mechanism contributing to increased PDAC risk by disturbing cell cycle progression via targeting cell cycle-related genes. To address this issue, we compared the miR-371-5p expression profiles between PDAC tissues and ANPT. We here show that miR-371-5p expression was significantly upregulated in PDAC tissues compared with ANPT, which resulted in cell proliferation and increased tumor growth. Thus, miR-371-5p can prove to be a novel prognostic factor and therapeutic target for pancreatic cancer treatment.

## Materials and Methods

### Cell culture

The PDAC cell lines SW-1990 and Panc-1 were obtained from the Chinese Center for Type Culture Collection (Shanghai, China). Cells were maintained in RPMI-1640 medium supplemented with 10% fetal bovine serum and antibiotics (100 U/ml penicillin and 100 µg/ml streptomycin sulfate). The cells were grown in a humidified incubator at 37°C supplemented with 5% CO2.

### Cell proliferation Assay

MTT [3-(4, 5-dimethylthiazol-2-yl)-2, 5-diphenyltetrazoliumbr-omide] - based assay was performed to estimate the effect of miR-371-5p on PDAC cell proliferation. Cells were seeded into 96-well plates (5,000 cells/well in 200 µl medium) and incubated for 24 hrs at 37°C, 5% CO_2_. PDAC cells were transfected with miR-NC, miR-371-5p, miR-control, anti-miR-371-5p, NC-siRNA or ING1-siRNA using Lipofectamine 2000 Reagent (Life Technologies) for 1, 2, 3 and 4 days, respectively, according to manufacturers' instructions. Cells cultured with complete medium were used as blank control. At the end of culture, 20 µl of 5 mg/ml MTT (Sigma, USA) solution was added per well, and the cells were incubated for another 4 h at 37°C. Supernatants were removed and formazan crystals were dissolved in 150 µl of dimethylsulfoxide (Sigma, USA). Finally, optical density was determined at 490 nm using multi-microplate test system (POLARstar OPTIMA, Germany). In each assay, five parallel wells were made, and the results were collected as the mean of more than three independent experiments. Lysates were harvested for western blot analysis.

### Cell cycle analysis

To analyze cell cycle, DNA content per duplicate was analyzed using FACSort Cellquect software (BD Biosciences, USA). PDAC Cells were placed in 6-well plates overnight and transfected with miR-NC, miR-371-5p, miR-con, anti—miR-371-5p, NC-siRNA, or ING1-siRNA for 48 hours respectively.

At the end of culture, cells were fixed in 75% ice-cold ethanol overnight at 4°C. The fixed cells were stained with 50 µg/ml propidium iodide (PI) containing 50 µg/ml RNase A (DNase free) for 15 min at room temperature in the dark and analyzed by fluorescence-activated cell sorting (Becton Dickinson, USA). The cells were excited at 488 nm, and the emission was collected through a 630 nm filter. In total, 20,000 cells were collected from each sample. The cell cycle distribution was evaluated by calculating the proportion of cells in G0/G1, S, and G2/M stages. In each independent experiment, three parallel wells were made, and the procedures were carried out in triplicate. Data obtained were presented as mean ± SD.

### Luciferase reporter assays

Briefly, the fragments of the 3′ untranslated region (3′-UTR) of ING1 mRNA containing the putative (WT) were amplified by PCR from genomic DNA, and the PCR product was subcloned into a pGL3-control vector (Promega, USA) immediately downstream of the luciferase gene. To construct mutant pGL3-ING1 (MU), the QuickChange site-directed mutagenesis kit (Stratagene) was used to induce six point mutations in the UTR region of WT-pGL3-ING1. Plasmid DNA was sequenced for authenticity.

For the luciferase reporter assay, cells were cotransfected with 300 ng of pGL3-ING1-WT or pGL3-ING1-Mut constructs and pcDNA3-miR-371-5p or negative control using Lipofectamine 2000 (Invitrogen) according to the manufacturer's protocol. Each sample was cotransfected with 50 ng of pRL-TK plasmid expressing renilla luciferase (Promega, USA). Cells were collected 48 h after transfection and analyzed using the Dual-Luciferase Reporter Assay System (Promega, USA). Relative luciferase measurements were made 48 h after the transfection using the dual Luciferase Reporter Assay System (Promega, USA) using a luminescence counter. Transfections were done in duplicate and repeated at least 3 times in independent experiments. MiR-371-5p inhibitor, siING1 and their negative controls (NC) were purchased from Panagene Inc (Korean) and Santa Cruz (USA) respectively. Sequences were as follows: miR-371-5p inhibitor: 5′-TTTTAACATTGCACT-3′. Ad-ING1 adenovirus (Cat#:1601) and its control Ad-GFP adenovirus (Cat#:1700) were purchased from Vector Biolabs (USA).

### Quantitative RT-PCR

Total RNA was isolated using Trizol reagent (Invitrogen, USA). 2 µg total RNAs were reverse transcribed using the miR-371-5p specific reverse transcription primers (Ribobio, China) using poly-A polymerase based First-Strand Synthesis kit (TaKaRa Bio, Japan) following the manufacturer's protocol, and the relative expression of miR-371-5p was determined using SYBR Premix Ex Taq (Takara, China). U6 snRNA was used for normalization. The delta-Ct method was used to evaluate the analyses of real-time PCR data. In semi-quantitative RT-PCR, the end PCR products after certain number of cycles were electrophoresed in 2% agarose gels followed by staining with ethidium bromide, and photographed under UV transillumination. Primers for miR-371-5p: RT primer, 5′-GTCGTATCCAGTGCAGGGTCCGAGGTATTCGCACTGGATACGACAGTGCC-3′; Forward, 5′-GTCGTATCCAGTGCAGCCG-CCACTCAAACTGTGGGG-3′. Primers for U6 snRNA: RT primer, 5′-GGGTCCGAGGTGCACTGGATACGACAAAAT-ATGG-3′; Forward, 5′-TGCGGGTGCTCGCTTCGGCAGC-3′.

### Chromatin immunoprecipitation assay (ChIP)

ING1 binding to the promoter of miR-371-5p was tested using ChIP analysis. ChIP assay was performed using the EZ-ChIP kit from Millipore (Millipore, USA) according to the manufacturer's instructions. Briefly, about 3×10^6^ Panc-1, infected with either Ad-GFP-ING1 or Ad-GFP adenoviruses were crosslinked using 1% formaldehyde (BIO-RAD, USA) for 15 min at 37°C. Cells were harvested after quenching with 0.125 M glycine and lysed in ChIP lysis buffer (150 mM NaCl, 1% Triton X-100, 50 mM Tris (pH 8.0), 0.1% deoxycholate, 1 mM EDTA, 1 mM PMSF, 1 µg/ml pepstatin, 1 µg/ml aprotinin and 1 µg/ml leupeptin). Extracts were sonicated six times for 9 s each and lysates were spinned by centrifugation at 14000 rpm for 20 min at 4°C. Of this sample, 150 µl was used as input. The supernatants were immunoprecipiated with either anti-ING1 or with mouse IgG (negative control) at 4°C for 4 h, followed by protein G Sepharose (Sigma, USA) for 2 h at 4°C. The immunoprecipitates were sequentially washed with 1 ml of ChIP lysis buffer twice, ChIP lysis buffer with 500 mM NaCl twice and with LiCl/detergent solution (10 mM Tris-HCl, pH 8.0, 250 mM LiCl, 0.5% NP-40, 0.5% sodium deoxycholate, 1 mM EDTA) twice and finally with TE buffer (10 mM Tris and 1 mM EDTA, pH 8.0). The beads were eluted using 1% SDS and 0.1 M sodium bicarbonate solution. The input and the eluent samples were reverse-crosslinked using NaCl for 5 h at 65°C. The DNA from the samples was isolated by phenol–chloroform, followed by ethanol precipitation. Promoter binding was tested by PCR using primers spanning the upstream regions of miR-371-5p start sites, primer sequences: ChIP-ING1, F: 5′-AATGTTCACTGCCGCACTGT-3′; R: 5′-ACACCTATAATCCCTGC- TAC-3′; ChIP-neg-F: 5′- ACGGCCAACATGCTCAGG-3′; R: 5′- TGTTTGCAACTGCTGCGTTAG-3′.

### Western blot

The cells were lysed with 1% RIPA Lysis Buffer (50 mM Tris-HCl, pH 8.0,150 mM Sodium chloride, 1% NP-40, 0.5% sodium deoxycholate, 0.1% sodium dodecyl sulfate, 2 mM EDTA) containing 1× protease inhibitor cocktail (Roche) 48 h after transfection. The supernatants were collected, and protein concentration was determined using the BCA Assay Kit (Pierce). The protein samples were separated on 10% SDS-PAGE and then transferred to a PVDF membrane. The membrane was blocked with 5% non-fat milk, followed by an overnight incubation at 4°C with a indicated primary rabbit antibody. The membrane was washed three times in TBST and then incubated with a secondary antibody. Last, the bound antibody was detected by chemiluminescence with the ECL Detection Reagent (Pierce). The data were normalized to GAPDH or β-actin.

### *In vivo* pancreatic cancer xenograft model

Panc-1 cells from moderately differentiated PDAC were stably transfected with miR-371-5p or miR-NC control plasmid. The transfected cells were collected, suspended in 200 µl PBS (1×10^7^ cells), and were subcutaneously injected in 4 week-old male BALB/c nude mice (*n* = 10/group). The mice were maintained in a specific pathogen-free environment for 5 weeks and then sacrificed. The tumor volume *V* was calculated using its length *L* and width *W*, according to the equation *V* = 0.4*LW*2. The use of nude mice complied with the Guide for the Care and Use of Laboratory Animals and the study was approved by Animal Care and Use Committee of the Southern Medical University.

### Patients and tumor tissues

A total of 60 human pancreatic cancer tissues (PC) and matched normal adjacent pancreatic tissues (NP) were obtained during the surgery at Baoan Hospital (Shenzhen, China) between January 2008 and December 2010. The diagnosis was based on pathological evidence. Furthermore, an additional 15 pairs of PDAC and their control samples were collected from patients during surgical resections and stored in liquid nitrogen. All 75 patients provided written informed consent for the use of their tissues and the study protocol was approved by the Ethics Committee of the Southern Medical University.

### Statistical analysis

The associations of miR-371-5p expression with the clinical pathologic characteristics were analyzed using the Chi-square or Mann–Whitney tests. The survival curve was constructed using the Kaplan–Meier method and compared using the log-rank test. Groups were compared using a Student's *t*-test. Correlation analysis was performed using Spearman's correlation analysis. All probability values were analyzed using a two-tailed test, and Statistical significance was concluded at ^*^P<0.05, ^**^P<0.01, ^***^P<0.001; # represents no statistical significance. The analyses were performed using SPSS (version 17.0) software (SPSS Inc., USA). Data were expressed as the mean ± standard deviation (SD).

## Results

### Upregulation of miR-371-5p in pancreatic cancer tissues is associated with survival

**Figure 1:**
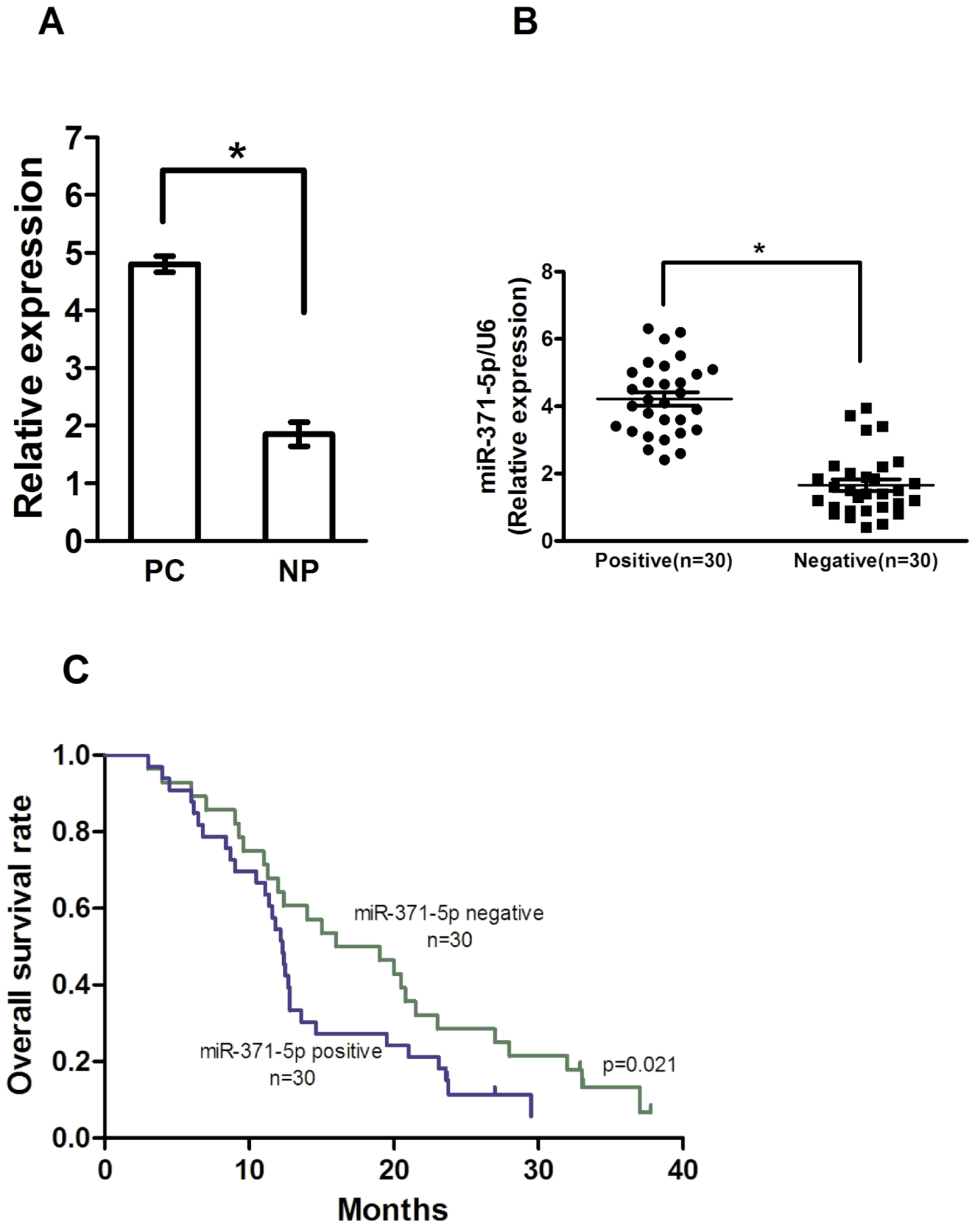
miR-371-5p expression in pancreatic cancer specimens and overall survival. (A) Average expression level of miR-371-5p in human PDAC specimens (*n* = 15) and normal pancreatic tissues (*n* = 15). miRNA abundance was assessed by qRT-PCR and normalized to U6 RNA. Values are presented as the mean ± S.D. (B and C) miR-371-5p expression levels and overall survival following resection of pancreatic cancer with the miR-371-5p -negative versus miR-371-5p -positive groups. The miR-371-5p - positive group had significantly shorter survival than the miR-371-5p - negative group (n = 30/group, *P* = 0.021).

To determine the expression levels of miR-371-5p in pancreatic cancer patients, miR-371-5p was detected in all the 15 pairs of pancreatic cancer tissues and their matched noncancerous pancreatic tissues using a qRT-PCR method. As shown in Fig.1A, PC tissues showed significantly higher expression of miR-371-5p as compared with that of the NP tissues (*P*<0.05). Moreover, the Kapan-meier survival analysis revealed that the patients with a miR-371-5p positive expression (Fig.1B) had a significantly poorer prognosis than those with a negative expression (Fig.1C). The multivariate Cox regression analysis indicated that miR-371-5p expression (hazards ratio [HR]  = 2.748, 95% confidence interval [CI]  = 1.211−5.914, P = 0.03), was independent prognostic factors for overall survival.

### MiR-371-5p promoted cell proliferation both *in vitro* and *in vivo*

**Figure 2:**
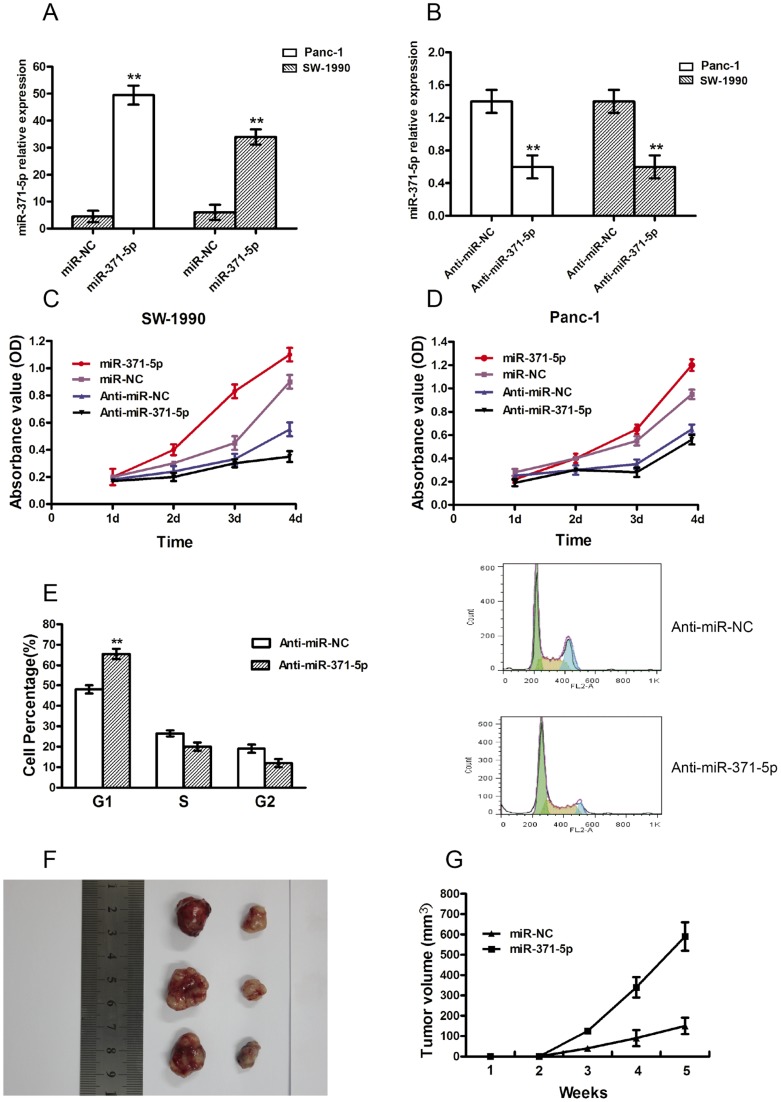
MiR-371-5p promoted the growth of pancreatic cancer cells *in vitro* and *in vivo*. (A and B) The expression level of miR-371-5p was tested for 48 h in pancreatic cancer cells transfected with miR-371-5p mimics, anti-miR-371-5p and their respective control (NC, 40 nM) by qRT-PCR. (C and D) The proliferation of PDAC cell lines transiently transfected with miR-371-5p mimics, miR-371-5p inhibitor or control was analyzed by the MTT proliferation assay. (E) Anti-miR-371-5p increases the proportion of cells in G1 as estimated by using flow cytometry (left panel, **P<0.01, n = 4). And representative cell cycle histographs (anti-miR-NC vs anti-miR-371-5p) are presented (right panel). (F) Nude mice were subcutaneously inoculated with Panc-1 cells transfected with miR-371-5p plasmid or miR-NC control plasmid, in their flanks. The image is representative of tumors formed in 10 mice. (G) Growth curves of tumor volumes. The graph is representative of tumor growth, 5 weeks after inoculation. Tumor volume was calculated and all data are shown as the mean ± S.D. (*n* = 10).

To explore the effect of miR-371-5p on pancreatic cancer cells growth, the PDAC cell lines with different grades SW-1990 and Panc-1 were transiently transfected with a miR-317-5p mimic or inhibitor (anti-miR-371-5p) and their respective NCs. The qRT-PCR analysis showed that the miR-371-5p mimics caused a 14.42 and 6.47-fold increase in the miR-371-5p expression in PANC-1 and SW-1990 cells, respectively (Fig.2A). Meanwhile, the anti-miR-371-5p decreased the miR-371-5p expression by 3.14 and 3.28 folds in PANC-1 and SW-1990 cells respectively, as compared with the control cell lines (Fig.2B). The MTT proliferation assay was conducted in SW-1990, and Panc-1 cells that were transiently transfected with a miR-371-5p mimic or inhibitor. The results showed that miR-371-5p -mimics promoted tumor cell growth, whereas the anti-miR-371-5p inhibited tumor cell growth (P<0.01, Fig.2C and D) To investigate the mechanism by which anti-miR-371-5 inhibits cell proliferation, we analyzed the cell cycle distribution of the Panc-1 and SW-1990 cells that were transfected with anti- miR-371-5p and the control. A larger proportion of cells transfected with the anti- miR-371-5p accumulated in the G1 phase, whereas the S phase population decreased (Fig.2E). However, an opposite result was observed in cells transfected with the miR-371-5p (data not shown). These results suggested that the proliferative inhibition of anti- miR-371-5p was partially due to a G1-phase arrest of the two pancreatic cancer cell lines.

To further determine whether miR-371-5p was involved in tumourigenesis, a stable miR-371-5p -overexpression cell line and control cell line were subcutaneously injected into the nude mice, and in turn, the tumour growth activity was measured. When the tumours were harvested, the average volume of the tumours derived from the miR-371-5p group was bigger compared with the control group (Fig.2F and G, *P*<0.05). These results were consistent with the effects of miR-371-5p overexpression in vitro and strongly suggested that miR-371-5p could promote the proliferation of PDAC cells.

### ING1 is a direct target of miR-371-5p and involved in miR-371-5p inducing promotion of PDAC cells proliferation

**Figure 3:**
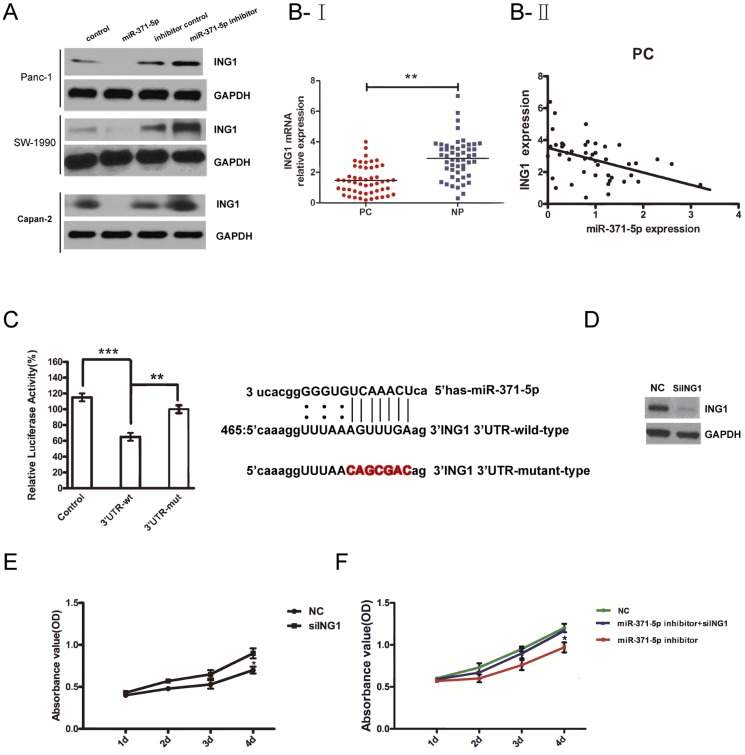
ING1 is one of the miR-371-5p targets and negatively regulated by miR-371-5p. (A) ING1 expression in SW-1990 and Panc-1 cells after transfection with anti- miR-371-5p, or miR-371-5p mimics, or control detected by western blotting. (B–I) ING1 was detected by qRTPCR in 50 pancreatic cancer (PC) tissues and was matched the adjacent noncancerous pancreatic tissues (NP). The ING1 abundance was normalized against GAPDH. (B-II)The relationship between ING1 and miR-371-5p expression was explored by Spearman's correlation in 50 PDAC tissues. ING1 expression was negatively correlated with miR-371-5p in 50 PDAC tumor tissues. (C) Predicted consequential pairing of target 3′-UTR region of ING1 (wild-type or mutated) and miR-371-5p mature sequence. Luciferase activity on the presence of both wild-type ING1 3′-UTR or mutant and miR-371-5p was compared with the control. (D) Western blot was performed to analyze ING1 expression in si ING1 transfected Panc-1 cells. β-actin was used as an internal quantitative control. (E) Effect of siING1 on cell proliferation was measured by MTS assay. (F) Panc-1 cells were transfected with NC, miR-371-5p inhibitor or miR-371-5p inhibitor and siING1, and then MTS assay was performed.

To explore the potential target through which the miR-371-5p promotes the proliferation of pancreatic cancer cells, we took advantage of bioinformatics to predict the putative miR-371-5p targets. Both Target Scan and miRanda systems predicted ING1, a key tumor suppressor, to be a putative target of miR-371-5p. To verify whether miR-371-5p directly targeted miR-371-5p mRNA in PDAC tumors and cell lines, in vitro and in silico analysis were conducted. First, anti- miR-371-5p or control was transfected into SW-1990 and Panc-1 cells, and we found that inhibited expression of miR-371-5p increased ING1 protein levels in both lines (Fig. 3A). Then, miR-371-5p mimic or control was transfected into the above 2 cell lines. We found that forced expression of miR-371-5p decreased ING1 protein levels in both cell lines consistently (Fig. 3A). These results indicated that miR-371-5p might promote the proliferation of PDAC cells with decreasing of ING1 expression in vitro.

To further confirm the regulatory relationship between miR-371-5p and ING1, we examined the expression of ING1 in PDAC samples (PC) and their corresponding normal tissues (NP). Using qRT-PCR, we found that the average level of ING1 expression was significantly lower in PC tissues as compared to that of the NP tissues (Fig. 3B–I). A significant inverse correlation was observed between ING1 and miR-371-5p expressions in pancreatic cancer tissues (Spearman's correlation, r = −0.4802) and adjacent noncancerous tissues (r = −0.4103, data not shown) (Fig. 3B-II).

To evaluate the ability of miR-371-5p binding to the 3′UTR of ING1, we performed a luciferase reporter assay and observed a significant decrease in luciferase activity in the presence of miR-371-5p - ING1 in SW-1990 cell, compared with the controls (Fig. 3C). To validate whether ING1 was a direct target of miR-371-5p, we mutated the miR-371-5p binding sites in the 3′UTR of ING1 and observed the loss of repression (Fig. 3C). Taken together, the data suggested that ING1 was a direct target of miR-371-5p.

To elucidate whether the growth-promoting effect of miR-371-5p was mediated by repression of ING1 in PDAC cells, the effect of ING1 on cell growth was examined. First, Panc-1 cells were transfected with siRNA against ING1 or its control, and then we examined cell growth by MTT assay (Fig. 3D). MTT assay results showed that gene silencing of ING1 promoted cell proliferation of Panc-1 cells (Fig.3E). Moreover, the inhibitory effect of miR-371-5p inhibitor on cell growth was reversed when ING1 expression was knockdown (Fig.3F). Taken together, these findings indicated that ING1 is a functionally important target of miR-371-5p which is involved in the proliferation of PDAC cells.

### ING1b regulates miR-371-5p expression

**Figure 4:**
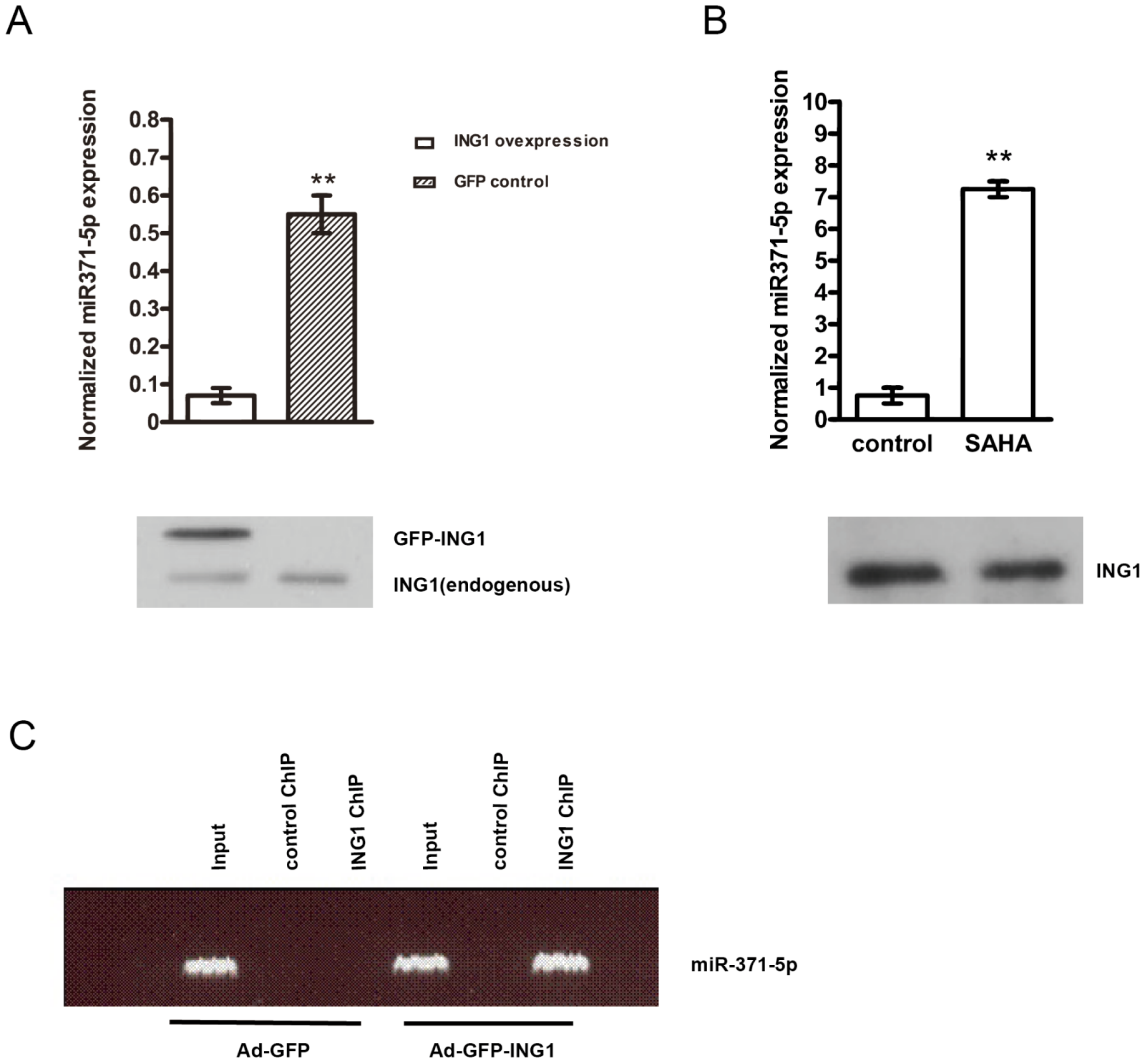
ING1b regulates miR-371-5p expression. (A) The expression of miR-371-5p in Panc-1 cells in response to ING1 overexpression for 48 hs, was determined by quantitative real-time PCR assays (upper panel. Bars represent the mean ± SD (**P<0.01, n = 5). (Lower panel) Western blot was performed to analyze ING1 expression. (B) miR-371-5p level was determined using real-time RT-PCR in Panc-1 cells in response to treatment with the HDAC inhibitor suberoylanilide hydroxamic acid (SAHA). Bars represent the mean ± SD (upper panel, *P<0.05, n = 5). (Lower panel) Western blot was performed to analyze ING1 expression. (C) Cells infected with control GFP adenovirus or with the same virus also encoding ING1 were harvested 24 h later and prepared for ChIP analysis using control or ING1 antibodies. PCR of immunoprecipitation products showed that ING1 bound to the upstream region of miR-371-5p.

The ING family of type II tumour suppressors serve as both epigenetic ‘readers’ and target histone acetyl transferase (HAT) and histone deacetylase (HDAC) ‘writers’ of the epigenetic histone code [11]. The ING1 protein has also been implicated in regulating miRNA levels [12]. To identify whether ING1 regulates miR-371-5p expression, Panc-1 cells were infected with adenovirus expressing GFP alone or with adenovirus expressing both GFP and ING1. MiR-371-5p was confirmed to decrease significantly and reproducibly in response to ING1 overexpression (Fig.4A). In addition, as shown in Fig.4B, suberoylanilide hydroxamic acid (SAHA), a histone deacetylase (HDAC) inhibitor, increased miR-371-5p expression significantly in Panc-1 cells, which is consistent with epigenetic regulation by ING1 as part of HDAC1 and HDAC2 complexes. However, SAHA did not change ING1 expression. ChIP analysis of ING1 showed that ING1 bound to the promoter area of miR-371-5p (Fig.4C), indicating that ING1 directly regulates miR-371-5p expression. Thus, ING1 epigenetically regulates miR-371-5p expression in PDAC.

## Discussion

A complete understanding of the molecular mechanisms underlying pancreatic tumor initiation and progression is important for novel prognostic and therapeutic approaches aimed at improving the outcome of patients with cancer. Over the last years, miRNAs are emerging as a new class of gene regulators involved in different tumors [13]. Here, we provide the first evidence that miR-371-5p plays a critical role in pancreatic tumorigenesis. It is frequently upregulated in PDAC. Consistently, miR-371-5p overexpression is markedly associated with adverse patients' survival, suggesting that it is a poor prognostic factor. The positive relationship of miR-371-5p levels with proliferation supports the notion that it acts as an onco-miRNA.

The characteristics observed in patients with PDAC are recapitulated in mouse xenograft models. MiR-371-5p-overexpressing cells develop large tumors, which are reverted by injections of a specific inhibitor against it. An opposite behavior is observed with miR-371-5p low-expressing cells and injection of the corresponding mimic RNA. To date, miR-371-5p has been shown to be involved only in hepatocellular carcinomas, which indicates that its overexpression is associated with more aggressive tumors and a poorer outcome [14], in agreement with data obtained in PDAC. However, the contribution of miR-371-5p to this process needs further investigations.

Our understanding of the biological functions of miRNA relies on the identification of relevant target genes. However, the prediction of miRNA targets is still a challenging research area. The majority of the miRNA-target sequence matching is based on imperfect complementarity of the miRNA with the 3′-UTRs of target mRNAs. The requirement for complementarity of only seven or eight nucleotides could result in hundreds of possible targets [15]. So far, very few target genes of miR-371-5p have been confirmed in different biological systems.

Herein, we validate ING1 as a direct functional target of miR-371-5p in PDAC, adding information to previously reported cell types [16]. The ING family of type II tumour suppressors contributes to the growth of various tumours [17]. This family is well conserved and includes five genes. Among them, *ing1* is the best characterized member and encodes four protein isoforms [18]. The ING family of tumor suppressors acts as readers and writers of the histone epigenetic code, affecting DNA repair, chromatin remodeling, cellular senescence, cell cycle regulation and apoptosis [18]. Overexpression of ING1 caused cell cycle arrest at G1 phase with ensuing apoptosis, whereas suppression of its expression increased colony focus formation and growth *in vitro* and tumour formation *in vivo* [19]. However, the detailed molecular mechanisms by which ING1 acts as a tumour suppressor remain incompletely defined.

ING1 regulates gene expression via regulating histone acetylation and methylation [20]. ING1 is the major target of the HDAC inhibitor SAHA, and appears to primarily regulate chromatin compaction and subsequently, the expression of subsets of genes through epigenetic mechanisms. Additionally, many miRNAs have been reported to be regulated by epigenetic mechanisms [21]. Therefore, we asked whether miR-371-5p is regulated by ING1. In the current study, we show that the expression of miR-371-5p is epigenetically regulated by ING1 and that miR-371-5p reverses a significant proportion of the inhibitory effects of ING1 on cell proliferation. Moreover, changes in ING1 expression affect cell proliferation and apoptosis, reproducing in an inverse mode the effects due to miR-371-5p, as further demonstrated in tumor samples. The reciprocal and inverse variations of ING1 levels following miR-371-5p modulation clearly demonstrate that they are mechanistically linked. The tight interplay between ING1 and miR-371-5p is further proven by their concomitant silencing: ING1 knockdown completely reverses the effects on cell proliferation and apoptosis due to miR-371-5p inhibition, identifying it as a major downstream effector of this miRNA. Thus, a potential mechanism for ING1-mediated G1 cell cycle arrest could involve the inhibition of miR-371-5p, further establishing links between miRNAs and the ING1 tumour suppressor. More experiments are required for a further understanding of the complex interplay between ING1 and tumorogenesis.

Given the present data that the inverse association of miR-371-5p with ING1 persists in PDAC, we hypothesize that the miR-371-5p-ING1 axis can significantly influence the progression. Targeting ING1 and miR-371-5p signaling pathways could then represent a rational approach for the treatment of PDAC. In this context, modulating miR-371-5p could restore ING1 expression sensitizing the tumor to a combined therapy of ING1 agonists and chemotherapeutic drugs routinely employed into the clinical practice.

In summary, we report the novel observation that miR-371-5p is frequently upregulated in PDAC and correlates with poor prognosis. These characteristics are reproduced in mouse xenografts of overexpressing or down-expressing miR-371-5p cells, and reverted by injections of miR-371-5p inhibitor or mimic. We show that ING1 silencing phenocopies miR-371-5p overexpression and provide mechanistic evidence that it is a major downstream mediator. A better understanding of the miR-371-5p-ING1 axis functions and interactions with other signaling pathways may clarify its clinical relevance and its prognostic role in pancreatic tumorigenesis. In light of the effective nature of targeting this pathway shown in this study, we propose that this strategy may provide an effective therapeutic approach for PDAC patients who have exhausted other modes of treatment.
